# Haemostatic differences between SARS-CoV-2 PCR-positive and negative patients at the time of hospital admission

**DOI:** 10.1371/journal.pone.0267605

**Published:** 2022-04-28

**Authors:** B. de Laat, M. J. M. Traets, R. W. M. De Laat-Kremers, S. P. Verweij, M. Ninivaggi, E. Jong, D. Huskens, B. A. Blok, G. C. P. Remme, A. Miszta, R. H. T. Nijhuis, G. J. M. Herder, R. Fijnheer, M. Roest, A. T. L. Fiolet, J. A. Remijn

**Affiliations:** 1 Synapse Research Institute, Maastricht, the Netherlands; 2 Department of Internal Medicine, Meander Medical Center, Amersfoort, the Netherlands; 3 Department of Medical Microbiology and Medical Immunology, Meander Medical Center, Amersfoort, the Netherlands; 4 Department of Pulmonology, Meander Medical Center, Amersfoort, the Netherlands; 5 Department of Clinical Chemistry, Meander Medical Center, Amersfoort, the Netherlands; King Faisal University, SAUDI ARABIA

## Abstract

Coronavirus disease 2019 (COVID-19), caused by severe acute respiratory syndrome coronavirus 2 (SARS-CoV-2) is associated with thrombosis. We conducted a cohort study of consecutive patients, suspected of SARS-CoV-2 infection presented to the emergency department. We investigated haemostatic differences between SARS-CoV-2 PCR positive and negative patients, with dedicated coagulation analysis. The 519 included patients had a median age of 66 years, and 52.5% of the patients were male. Twenty-six percent of the patients were PCR-positive for SARS-CoV-2.PCR positive patients had increased levels of fibrinogen and (active) von Willebrand Factor (VWF) and decreased levels of protein C and α2-macroglobulin compared to the PCR negative patients. In addition, we found acquired activated protein C resistance in PCR positive patients. Furthermore, we found that elevated levels of factor VIII and VWF and decreased levels of ADAMTS-13 were associated with an increased incidence of thrombosis in PCR positive patients. In conclusion, we found that PCR positive patients had a pronounced prothrombotic phenotype, mainly due to an increase of endothelial activation upon admission to the hospital. These findings show that coagulation tests may be considered useful to discriminate severe cases of COVID-19 at risk for thrombosis.

## Introduction

The coronavirus disease 2019 (COVID-19), caused by severe acute respiratory syndrome coronavirus 2 (SARS-CoV-2) infection [1–3], has a high mortality in particular for elderly and patients with pulmonary or cardiovascular comorbidities [4–6]. The clinical course of COVID-19 varies from asymptomatic or mild flu-like symptoms to severe pneumonia, respiratory failure or even acute respiratory distress syndrome (ARDS) [7, 8]. Around 30% of the hospitalized patients with severe symptoms of COVID-19 require admission to the intensive care unit (ICU) of whom 30–40% develop ARDS [9]. Mortality of hospitalized patients with COVID-19 is estimated at 5% [9–11].

COVID-19 is associated with a prothrombotic phenotype with an increased risk for thrombosis [4, 12]. Several studies have shown thrombotic complications varying from 23% to 69% in ICU patients with COVID-19 [13–16]. Haemostatic abnormalities such as prolonged prothrombin time, prolonged activated partial thromboplastin time (aPTT), decreased levels of antithrombin and fibrinogen, increased fibrin degradation products and D-dimer are common in severe COVID-19 suggesting that conventional coagulation may play an important role in COVID-19 disease [2]. A pro-coagulant phenotype is associated with a more severe course of COVID-19 and an increased risk of death [10, 17]. Hospitalized COVID-19 patients are treated with low molecular weight heparin (LMWH) to prevent development of thrombosis such as pulmonary embolism or stroke [13, 18]. Despite treatment with LMWH in prophylactic or therapeutic dose, thrombotic complications such as disseminated intravascular coagulopathy (DIC) still occur. The aim of this study was to investigate whether COVID-19 is associated with changes in coagulation parameters during presentation at the emergency department and whether these changes are associated with the development of thrombotic complications in patients with SARS-CoV-2 infection.

## Material and methods

### Study design and population

We conducted a single center, cross-sectional cohort study: the MArkers in COVID-19 And Relations to Outcomes in the Netherlands (MACARON) study. The study was performed in accordance with the Declaration of Helsinki. Approval was obtained from the local Medical Ethical Committee (TWO 20–043). The need for informed consent was waived by the ethics committee because of urgency of data collection in patients with a new disease with suspected increased risk of thrombosis. Plasma remnants of routine blood drawing were used for laboratory testing and patient samples were analyzed completely anonymously. Patients were not subjected to invasive procedures and only additional experimental laboratory assays were performed.

All patients suspected of SARS-CoV-2 infection referred to the emergency department of the Meander Medical Center in Amersfoort between 23 March and 7 May 2020 were included in the study. Blood samples were obtained from all patients upon presentation at the emergency department and stored at—80 degrees Celsius until further analysis. Both an oro- and nasopharyngeal swab were obtained for the detection of SARS-CoV-2 by real-time polymerase chain reaction (RT-PCR) targeting the E-gene as described by Corman *et al*. [19]. A test was defined as SARS-CoV-2 positive when a signal with cycle threshold (Ct-) value of <45 was found, showing a correct sigmoid curve.

Patients were divided into two groups based on PCR results: positive or negative. Clinical data were collected using an electronic case report form based on a template of the World Health Organization [20]. Demographics (gender, age, race) and clinical data (comorbidities, medication use) were extracted from electronic heath care records. Ethnicity was determined based on family name. Comorbidities and medication use were collected as present at the time of emergency department visit.

### Outcomes

Thrombosis was defined as a clinical diagnosis of venous thromboembolism (pulmonary embolism, deep vein thrombosis) or atherothrombotic event (acute coronary syndrome, cerebral ischemic attack or mesenteric ischemia). Diagnoses were made to the discretion of the treating physician by the use of computed tomography, ultrasonography, or electrocardiogram and laboratory results when appropriate. Mortality was noted if a patient died during hospitalization or <14 days after palliative discharge.

### General laboratory tests

Blood gas analysis was performed using the RapidPoint 500 analyzer (Siemens, Germany). Complete blood cell count was measured by Sysmex XN-9000 (Sysmex, Germany). Prothrombin Time (PT) and aPTT were measured by Sysmex CS-5100 using Innovin and Actin FSL reagents (Siemens, Germany), respectively. C-Reactive Protein (CRP) and liver parameters (ALAT, ASAT, GGT) were measured by Architect C16000 (Abbott, USA). Ferritin was measured by Architect i2000SR (Abbott, USA).

### Coagulation factor analysis

Plasma levels of antithrombin, fibrinogen, FVIII, protein C and D-dimer levels were measured in citrated plasma on the STA-R Max according to the manufacturer’s recommendation (Diagnostica Stago, France). Functional α_2_-macroglobulin (α_2_M) levels were measured as previously described [21]. ADAMTS-13 levels were measured in a FRET assay (Biomedica Diagnostics, Canada).

### Assay to detect active von Willebrand Factor (VWF), total VWF and VWF propeptide

Total VWF antigen and active VWF levels were quantified using in-house developed enzyme linked immunosorbent assay (ELISA), as described previously [22]. Briefly, 96 well microtiter plates (NUNC Maxisorp, Thermo Fisher Scientific, Waltham, USA) were coated overnight at 4°C with 1.98 μg/ml with a VHH antibody against active VWF (A1 domain) or 0.775 μg/ml rabbit anti-VWF polyclonal antibody (A0082; Dako) in a carbonate-bicarbonate coating buffer (pH 9.6), followed by a blocking step with 2% BSA in phosphate-buffered saline (PBS) for 45 minutes at room temperature. After washing with 0.01% Tween-20 in PBS, plasma samples (diluted 1:20 and 1:160 in PBS/1% BSA to detect active VWF and total VWF, respectively) were incubated for 2 hours at room temperature. Following another wash-step, wells were incubated with HRP-conjugated anti-VWF polyclonal antibody (1.2 μg/mL; P0226, Dako) in PBS/1% BSA for 2 hours at room temperature. Plates were then washed again before addition of SIGMAFAST OPD (Sigma). The reaction was stopped with 2 M sulfuric acid (H2SO4, Sigma). Optical densities were measured at 490 nm using an ELx808 Absorbance Microplate Reader (Biotek, Bad Friedrichshall, Germany). VWF propeptide was measured with an ELISA, using the MW1939 antibody pair and Tool Set 2 (Sanquin, Amsterdam, The Netherlands), according to the manufacturer instructions. The level of VWF propeptide, active VWF and total VWF were expressed as a percentage of the level in normal pooled plasma included on the same plate.

### Thrombin generation

Thrombin Generation (TG) was measured in citrated plasma by Calibrated Automated Thrombinography (CAT) using PPP reagent (Diagnostica Stago, France). Thrombin generation was measured at 5 pM tissue factor in the presence and absence of thrombomodulin (TM; the concentration causing 50% inhibition of the peak height in pooled normal plasma; Synapse Research Institute, The Netherlands) to test the sensitivity of the Activated Protein C anticoagulant pathway.

### Plasmin generation assay

Plasmin generation (PG) was measured in human plasma using a calibrated automated method previously developed for thrombin generation assay [23]. This assay is based on cleavage of a plasmin-specific fluorogenic substrate and calibration with α_2_-macroglobulin-plasmin complex (α_2_M-Pm) [23]. To perform PG, first 35 μL of plasmin generation trigger (Synapse Research Institute, Maastricht, Netherlands) containing tissue factor, phospholipids and recombinant tissue plasminogen activator (final concentrations 0.5 pM, 4 μM, 1.25 μg/mL, respectively) were added to the first well and 35 μL α_2_M-Pm calibrator (Synapse Research Institute, Maastricht, Netherlands) to the second well. To study the effects of thrombomodulin, plasmin generation was measured with and without thrombomodulin (TM; 2 nM final). Next 15 μL of plasma was added to each well and plates were heated for 10 minutes at 37°C in fluorometer (Fluoroskan Ascent, Thrombinoscope, Maastricht, the Netherlands). Reactions were initiated by dispensing to both wells 10 μL of fluorogenic substrate solution with CaCl_2_ (0.5 mM and 16.6 mM, final concentrations respectively). Reactions were monitored every 20 seconds with a fluorometer equipped with a dispenser and 390/460 filter set (excitation/emission). Data were analyzed as previously described to correct for substrate consumption during reaction and inner filer effect [24]. Parameters obtained from PG assay were lagtime (time the plasmin concentration reached 6 nM), TtPeak, velocity (peak/[TtPeak-lagtime]), peak, and endogenous plasmin potential (EPP).

### Statistical analysis

Statistical analyses were performed using IBM SPSS Statistics for Windows, version 26 (IBM Corp., Armonk, N.Y., USA). Patient characteristics and outcomes were analyzed using descriptive statistical methods and presented as number (%), mean (standard deviation [SD] or standard error of the mean (SEM)) or median (interquartile ranges [IQR]). Differences between group means were tested with a Mann Whitney U test for continuous variables and with a Chi square test for categorical variables. A p-value of <0.05 was considered statistically significant.

## Results

A total of 591 patients suspected of SARS-CoV-2 infection presented to the emergency department of the Meander Medical Center in the Netherlands. Five patients did not meet the inclusion criteria as they were younger than 18 years and were excluded from the study. Subsequently, 586 patients were included in the study. Of those, 519 (87.8%) were included in the analysis. Sixty-seven patients (12.9%) were excluded from analysis: either due to no nasopharyngeal swab taken (n = 38), or insufficient plasma sample material (n = 29). Of the 519 patients, 135 (26.0%) had a positive SARS-CoV-2 PCR result. All hospitalized patients had complete follow-up until discharge or death.

### Baseline characteristics

**Table 1** shows baseline characteristics of the PCR positive and PCR negative patients. The proportion of males compared to females was significantly higher in PCR positive patients compared to PCR negative patients (66.6% male vs. 47.1% male, P < 0.001), whereas age distribution was comparable between the two groups (64 years [IQR: 55–75 years] vs. 57 years [IQR:53–78 years]). PCR positive patients were more often non-Caucasian than PCR negative patients (14.1% versus 7.3%, P = 0.018). There were no differences between PCR positive and PCR negative patients in presence of pulmonary or cardiovascular comorbidities. PCR positive patients used less frequently anticoagulation with direct oral anticoagulant or antithrombotic agents.

**Table 1:** Baseline characteristics of PCR SARS-CoV-2 negative and positive patients.

			PCR-negative (N = 384)		PCR-positive (N = 135)		p-value
Age, years			57	(53–78)	64	(55–75)	ns
Male gender, N (%)			181	(47.1)	45	(66.6)	<0.001
Caucasian race, N(%)			356	(92.7)	116	(85.9)	0.018
Body-mass index ≥ 30 kg/m^2^, N							
			90	(26.8)	25	(20.5)	ns
Comorbidities, N (%)							
	Pulmonary disease		145	(37.8)	43	(31.9)	ns
	Coronary artery disease		76	(19.8)	23	(17.0)	ns
	Auto-immune or inflammatory disease		70	(18.2)	18	(13.3)	ns
	Diabetes Mellitus		78	(20.4)	21	(15.6)	ns
	Prior venous-thromboembolism		29	(7.6)	5	(3.7)	ns
	Active malignant neoplasm		35	(9.1)	5	(3.7)	ns
Medications at baseline, N (%)							
	Antithrombotic/anticoagulant agents		171	(44.5)	42	(31.1)	0.006
		Antiplatelet agent	97	(25.3)	28	(20.7)	ns
		Direct oral anticoagulants	52	(13.5)	9	(6.7)	0.033
		Coumarin	32	(8.3)	7	(5.2)	ns
	ACE inhibitor or ARB		92	(24.0)	35	(25.9)	ns

All variables except age are reported as the number of cases (N) with the percentage of the respective group between brackets. Age is reported as median with the interquartile range between brackets. ACE, angiotensin-converting enzyme; ARB, angiotensin II receptor blocker.

### Clinical outcomes

Of the PCR positive patients 113/135 (83.7%) were hospitalized with a median duration of admission of 5 days (IQR 3–12 days). Of the hospitalized patients 28/113 (24.8%) were admitted to the ICU. Thrombotic complications during follow-up occurred significantly more often in PCR positive patients as compared to PCR negative patients (14.4% vs. 5.1%, p = 0.001) (**Table 2**). Mortality was significantly higher in PCR positive patients than PCR negative patients (15.3% vs. 7.6%, p = 0.015). This effect was attributed to a higher mortality in the female subset of the PCR positive compared to PCR negative group (14.0% vs 4.2%, p = 0.016). No difference in mortality was found between male PCR-positive or -negative patients (16.4% vs. 10.4%).

**Table 2:** General laboratory tests and specific coagulation test in PCR SARS-CoV-2 negative and positive patients.

	References	PCR-negative (n = 384)	PCR-positive (n = 135)	p-value
Thrombosis during follow-up (%)		5.1%	14.4%	0.001
Mortality during follow-up (%)		7.6%	15.3%	0.015
O2 saturation (%)	0.94–0.99	0.921 (±0.001)	0.921 (±0.001)	ns
PO2 (kPa)	9.3–13.3	9.11 (±0.21)	8.41 (±0.21)	0.022
PCO2 (kPa)	4.7–6.0	4.91 (±0.11)	4.21 (±0.11)	<0.001
pH	7.35–7.45	7.41 (±0.01)	7.51 (±0.01)	<0.001
Platelets (*10^9/L)	150–350	263 (±5)	227 (±8)	<0.001
Erythrocytes (*10^12/L)	4.0–5.5	4.41 (±0.01)	4.61 (±0.11)	0.029
Leukocytes (*10^9/L)	4.5–11.0	11.1 (±0.1)	8.1 (±1.1)	<0.001
Lymphocytes (*10^9/L)	0.6–4.8	1.91 (±0.31)	1.01 (±0.11)	<0.001
Neutrophils (*10^9/L)	1.5–8.0	8.41 (±0.31)	5.51 (±0.31)	<0.001
ALAT (IU/L)	5–45	42.1 (±6.1)	43.1 (±5.1)	<0.001
ASAT (IU/L)	5–35	51.1 (±7.1)	61.1 (±5.1)	<0.001
Gamma-GT (IU/L)	5–55	72.1 (±7.1)	75.1 (±6.1)	<0.001
Ferritin (μg/L)	20–250	386 (±46)	1466 (±153)	<0.001
CRP (mg/L)	0–5	69.1 (±5.1)	116.1 (±8.1)	<0.001
PT (sec)	10.0–12.0	13.1 (±0.1)	13.1 (±1.1)	ns
APTT (sec)	25–33	28.1 (±0.1)	31.1 (±1.1)	<0.001
D-dimer (μg/mL)	0.01–0.51	1.71 (±0.11)	1.91 (±0.21)	ns
Fibrinogen (g/L)	1.81–4.51	4.21 (±0.11)	5.41 (±0.21)	<0.001
Protein C (%)	65–135	96.1 (±2.1)	85.1 (±2.1)	<0.001
Antithrombin (%)	98–137	96.1 (±1.1)	100.1 (±1.1)	0.032
α2-macroglobulin (μM)	1.71–4.71	5.11 (±0.11)	4.41 (±0.21)	<0.001
VWF (%)	50–200	156 (±3)	189 (±4)	<0.001
active VWF (%)	92–155	140 (±4)	157 (±6)	<0.001
VWF propeptide (%)	73–189	224 (±8)	221 (±9)	ns
FVIII (%)	76–237	182 (±5)	168 (±7)	ns
Thrombin generation:				
ETP (nM·min)	899–1697	1208 (±22)	1256 (±37)	ns
Peak (nM)	185–462	198 (±5)	213 (±8)	ns
Lag time (min)	1.71–3.81	4.41 (±0.11)	4.51 (±0.21)	0.020
Time-to-peak (min)	3.21–6.61	8.21 (±0.21)	7.91 (±0.31)	ns
Velocity index (nM/min)	55–289	69.1 (±2.1)	79.1 (±5.1)	ns
Time-to-tail (min)	4.8–30.9	24.1 (±0.1)	24.1 (±1.1)	ns
Curve width (min)	12.8–27.7	21.1 (±0.1)	21.1 (±1.1)	ns
Decay index (nM/min)	39–124	51.1 (±2.1)	54.1 (±3.1)	ns
Peak inhibition by TM (%)	20–73	24.1 (±1.1)	17.1 (±2.1)	<0.001
ETP inhibition by TM (%)	11–68	37.1 (±1.1)	31.1 (±2.1)	0.009
Plasmin generation:				
EPP (nM·min)	237–535	610 (±18)	776 (±40)	<0.001
Plasmin Peak (nM)	82–132	113 (±2)	123 (±3)	0.001
Plasmin Lag time (min)	3.31–8.01	5.41 (±0.21)	5.21 (±0.21)	0.005
Plasmin Time-to-peak (min)	5.01–9.71	7.61 (±0.21)	7.71 (±0.21)	<0.001

Results are indicated as mean and standard error. Results are indicated as mean and standard error. pO2, partial pressure of oxygen; pCO2, partial pressure of carbon dioxide; PT, prothrombin time; APTT, activated partial thromboplastin time; ALAT, alanine aminotransferase; ASAT, aspartate aminotransferase; ETP, endogenous thrombin potential; TM, thrombomodulin; EPP, endogenous plasmin potential; VWF, von Willebrand Factor; FVIII, factor VIII.

### General laboratory testing

**Table 2** shows general laboratory tests at presentation in relation to the reference intervals.

Blood gas analysis showed significantly decreased partial oxygen and carbon dioxide pressures and increased pH levels in PCR positive patients. Platelets, total leukocytes and lymphocyte counts were lower in PCR positive patients. Additionally, acute phase proteins were above normal levels in both groups, and significantly higher in PCR positive patients than PCR negative patients (C-Reactive protein, 116.1 mg/L vs 69.1 mg/L, P < 0.001, Ferritin 1466 μg/L vs. 386 μg/L, P < 0.0001). General coagulation analysis revealed significantly prolonged aPTT in PCR positive patients compared to PCR negative patients (31.1 seconds vs. 28.1 seconds, P <0.001). Additionally, fibrinogen levels were increased in PCR positive patients (5.41 g/L vs. 4.21 g/L, p < 0.001), which is in line with the increase in other acute phase proteins CRP and ferritin). D-dimer levels were above the normal range in both groups, and the D-dimer level did not differ between the PCR-negative as PCR-positive group.

### Specific hemostasis testing

The total thrombin production (ETP) and speed of thrombin production (thrombin peak and velocity index) were within the normal range and did not differ between PCR positive patients and PCR negative patients. The time-dependent variables lag time and time-to-peak were prolonged compared to the normal range and the lag time was significantly prolonged in PCR positive patients compared to PCR negative patients (4.51 min vs. 4.41 min, P = 0.020). Other variables related to thrombin generation such as the decay index, time-to-tail, and the curve width (time-to-tail–lag time) did not differ significantly between PCR positive patients and PCR negative patients. Adding thrombomodulin to test the activated protein C dependent inhibition of both factors V and VIII showed less effect in PCR positive patients than in PCR negative patients (17.1% vs. 24.1%, P < 0.001), indicating a certain level of acquired activated protein C resistance.

Several markers of the inhibitory pathways of coagulation were decreased: Protein C as inhibitor of factor V and VIII was lower in PCR positive patients compared to PCR negative patients (85.1% vs. 96.1%, P < 0.001), similar to α_2_M as inhibitor of FIIa levels (4.41 μM vs. 5.11 μM, P < 0.001). In contrast, we found that the antithrombin levels (inhibitor of FIIa) were slightly, but statistically significant increased in PCR positive patients (100.1% vs. 96.1%, P = 0.032), but in both groups within the normal reference range.

PCR positive patients showed signals of vascular system activation by means of elevated levels of total von Willebrand Factor (VWF) antigen (189% vs. 156%, P < 0.001) and active VWF (157% vs. 140%, P < 0.001). This did not lead to higher FVIII activity levels. Propeptide levels of VWF were comparable between groups, but elevated compared to the normal ranges.

Fibrinolysis as expressed by the endogenous plasmin potential (EPP) was increased in PCR positive patients compared to PCR negative patients (776 nM·min vs 610 nM·min, P <0.001). Analogous, the plasmin peak, defined as the maximum speed of plasminogen into plasmin conversion, was also increased (123 nM vs. 113 nM, P = 0.005) and time-dependent parameters, such as lag time and time-to-peak, were shorter in PCR positive patients.

### Thrombosis

In the PCR-positive group 19 patients (14.4%) developed thrombosis versus 19patients (5.1%) in PCR negative patients. The median duration of hospitalization was 25 days (IQR 12–40 days) for the thrombosis group as compared to 5 days (IQR 3–9 days) for the non-thrombosis group. **Table 3** shows demographic, general laboratory testing and specific haemostatic testing of PCR positive patients with thrombosis and without thrombosis. Thrombosis was more common in males than in females. There was no difference in age. Mortality was higher in the non-thrombosis group than in the thrombosis group (16.1% vs 10.5%, P <0.001). PT, aPTT, D-dimer and fibrinogen levels did not differ significantly, but ferritin and CRP levels were significantly higher in the thrombosis group (ferritin 2193 μg/L vs 1320 μg/L, p<0.001 and CRP 172 mg/L vs 106 mg/L, P = 0.007). Coagulation inhibitors Protein C and antithrombin did not differ between PCR positive patients with thrombosis compared to those without thrombosis, but α_2_M was decreased in those with thrombosis (3.71 μM vs 4.51 μM, p = 0.045). There were no differences in thrombin or plasmin generation between both groups. VWF levels were significantly higher in patients with thrombosis (208% vs 186%, P = 0.038), as were FVIII levels (208% vs 162%, P = 0.028). Active VWF and VWF propeptide levels did not differ between PCR positive patients with or without thrombosis. ADAMTS-13 levels were significantly lower in PCR positive patients with thrombosis (597±27 ng/mL vs. 691±27 ng/mL, p<0.001).

**Table 3:** General laboratory tests and specific coagulation test in PCR SARS-CoV-2 positive patients with and without thrombosis.

	References	PCR-positive without thrombosis (n = 113)	PCR-positive with thrombosis (n = 19)	p-value
Gender (% male)	NA	63.7%	73.7%	0.028
Age (years)	NA	64.1 (±1.1)	61.1 (±2.1)	ns
Mortality (%)	NA	16.1%	10.5%	<0.001
O2 saturation (%)	0.94–0.99	0.921 (±0.001)	0.901 (±0.011)	ns
pO2 (kPa)	9.3–13.3	8.51 (±0.21)	7.91 (±0.51)	ns
pCO2 (kPa)	4.7–6.0	4.31 (±0.11)	4.11 (±0.11)	ns
pH	7.35–7.45	7.51 (±0.01)	7.51 (±0.01)	ns
Platelets (*10^9/L)	150–350	228 (±8)	225 (±18)	ns
Erythrocytes (*10^12/L)	4.0–5.5	4.61 (±0.11)	4.61 (±0.21)	ns
Leukocytes (*10^9/L)	4.5–11.0	8.61 (±1.41)	7.71 (±0.91)	ns
Lymphocytes (*10^9/L)	0.6–4.8	1.11 (±0.11)	0.91 (±0.11)	ns
Neutrophils (*10^9/L)	1.5–8.0	5.41 (±0.31)	6.31 (±0.81)	ns
ALAT (IU/L)	5–45	45.1 (±6.1)	31.1 (±4.1)	ns
ASAT (IU/L)	5–35	62.1 (±6.1)	51.1 (±4.1)	ns
Gamma-GT (IU/L)	5–55	76.1 (±7.1)	69.1 (±16.1)	ns
Ferritin (μg/L)	20–250	1320 (±167)	2193 (±345)	<0.001
CRP (mg/mL)	0–5	106 (±8)	172 (±24)	0.007
PT (sec)	10.0–12.0	13.1 (±1.1)	12.1 (±0.1)	ns
APTT (sec)	25–33	31.1 (±1.1)	29.1 (±1.1)	ns
D-dimer (μg/mL)	0.01–0.51	1.61 (±0.21)	3.41 (±1.31)	ns
Fibrinogen (g/L)	1.81–4.51	5.31 (±0.21)	6.11 (±0.41)	ns
Protein C (%)	65–135	85.1 (±2.1)	83.1 (±5.1)	ns
Antithrombin (%)	98–137	99.1 (±2.1)	105.1 (±3.1)	ns
α2-macroglobulin (μM)	1.71–4.71	4.51 (±0.21)	3.71 (±0.31)	0.045
VWF (%)	50–200	186 (±4)	208 (±9)	0.038
active VWF (%)	92–155	157 (±7)	162 (±10)	ns
VWF propeptide (%)	73–189	216 (±10)	249 (±23)	ns
FVIII (%)	76–237	162 (±7)	208 (±23)	0.028
ADAMTS13 (ng/mL)	401–931	691 (±27)	597 (±27)	<0.001
Thrombin generation:				
ETP (nM·min)	899–1697	1236 (±38)	1329 (±126)	ns
Peak (nM)	185–462	210 (±8)	232 (±23)	ns
Lag time (min)	1.71–3.81	4.41 (±0.21)	4.71 (±0.61)	ns
Time-to-peak (min)	3.21–6.61	7.81 (±0.31)	8.41 (±1.21)	ns
Velocity index (nM/min)	55–289	77.1 (±5.1)	89.1 (±13.1)	ns
Time-to-tail (min)	–14.8–30.9	24.1 (±1.1)	23.1 (±2.1)	ns
Curve width (min)	–12.8–27.7	21.1 (±1.1)	20.1 (±1.1)	ns
Decay index (nM/min)	–39–124	54.1 (±3.1)	58.1 (±6.1)	ns
Peak inhibition by TM (%)	20–73	17.1 (±2.1)	16.1 (±3.1)	ns
ETP inhibition by TM (%)	11–68	30.1 (±2.1)	31.1 (±4.1)	ns
Plasmin generation:				
EPP (nM·min)	237–535	751 (±41)	907 (±134)	ns
Plasmin Peak (nM)	82–132	124 (±3)	123 (±6)	ns
Plasmin Lag time (min)	3.31–8.01	5.21 (±0.21)	5.31 (±0.41)	ns
Plasmin Time-to-peak (min)	5.01–9.71	7.61 (±0.21)	8.11 (±0.61)	ns

Results are indicated as mean and standard error. Results are indicated as mean and standard error. ADAMTS13, A Disintegrin And Metalloproteinase with a Thrombospondin type 1 motif, member 13; APTT, activated partial thromboplastin time; ALAT, alanine aminotransferase; ASAT, aspartate aminotransferase; CRP, C-Reactive protein; ETP, endogenous thrombin potential; TM, thrombomodulin; EPP, endogenous plasmin potential; pO2, partial pressure of oxygen; pCO2, partial pressure of carbon dioxide; PT, prothrombin time; VWF, von Willebrand Factor; FVIII, factor VIII.

## Discussion

In this study we investigated a broad range of specific haemostatic parameters in SARS-CoV-2 PCR positive patients presenting at the emergency department during the first period of the SARS-CoV-2 pandemic. We found that SARS-CoV-2 PCR positive patients had a prothrombotic phenotype as seen by higher fibrinogen and VWF levels [25], and acquired activated protein C resistance [26]. One out of seven hospitalized PCR positive patients developed thrombosis, which corresponds well to prevalence of thrombosis in COVID-19 patients reported in other studies [27]. The patients that developed thrombosis had elevated levels of established risk factors for thrombosis such as higher VWF and FVIII levels and lower ADAMTS-13 levels.

Most laboratory variables in the PCR positive patients are in line with a biochemical prothrombotic phenotype, although there are some remarkable differences. For example, although antithrombin levels were slightly higher in the PCR positive group (albeit within the reference range), no significant differences were observed between PCR positive subjects with or without thrombosis. Furthermore, increased plasmin generation in PCR positive patients indicates that fibrinolysis is increased, which can be explained by a continuous activation of coagulation. In another study, we previously reported an increase of plasmin generation in COVID-19 patients, which is in line with our current results [28]. The difference might be due to the different onset of the studies.

Additionally, increased plasma VWF levels are an indicator of endothelial dysfunction, as the vascular endothelium is involved in the VWF production [29, 30]. Higher VWF levels were observed in PCR positive patients, and VWF pro-peptide levels were increased in all groups, indicating that the vascular endothelium has been activated. Elevated levels of VWF and indirectly increased levels of FVIII, which forms complexes with VWF in the blood, have been associated with thrombotic events caused by endothelial activation [30]. In addition, the reduced levels of ADAMTS-13, which is responsible for cleaving VWF into smaller and less active molecules [31], in the thrombosis group supports the implication of VWF and ADAMTS-13 in the pathogenesis of the typical SARS-CoV-2 prothrombotic phenotype [32]. Therefore, a potential target in the thrombophylactic treatment of COVID-19, could be the modulation of endothelial cell activation, in particular related to VWF and ADAMTS-13.

In addition to the changes in the patients coagulation profile, we observed the increase of inflammatory markers such as ferritin and C-reactive protein in SARS-CoV-2 PCR positive patients confirming previous reports [33]. Especially in the subset of patients that develop thrombosis, inflammatory markers are known to be increased [34, 35]. The coagulation system is known to interact with the immune system [35], and subjects in an inflammatory state are likely to exhibit a prothrombotic phenotype in COVID-19 infection and other diseases [35–37]. Interestingly, von Willebrand Factor and its regulator ADAMTS-13 have been implicated in vascular inflammation and the development of immunothrombosis [38], providing a potential causative link between inflammation and coagulation in SARS-CoV-2 patients.

We acknowledge several limitations of our study. Firstly, the study was conducted during the first months of the pandemic. At that current time, no evidence of immunomodulatory therapy such as dexamethasone was available and experimental treatment strategies such as the use of hydroxychloroquine were still applied. Secondly, we only included patients based on a positive PCR for SARS-CoV-2 in the initially obtained oro-/nasopharyngal swab. Varying sensitivity of the PCR in this type of specimen in comparison to a specimen obtained from the lower respiratory tract could have led to the misclassification of some of the patients. Thirdly, there is a risk for observational bias for venous thromboembolism, as diagnostic tests such as computed tomography are more likely to be performed in PCR positive patients compared to patients in routine clinical care. Fourthly, 519 patients with suspected OCVID-19 were enrolled in the study at the time of hospital admission, of which only 135 tested positive for SARS-CoV-2, and the other 384 hospitalized patients formed the control group for this study. In our opinion, the enrollment of patients at the time of hospital admission gives valuable clinical information, as this is the time at which clinical decisions are needed. Additionally, we compare COVID-19 positive patients admitted to hospital to control patients that are admitted to the hospital instead of healthy controls. Even though patient cohort is not very extensive, this does allow the comparison mortality and thrombosis rate of SARS-CoV-2 patients to other hospitalized controls.

In conclusion, SARS-CoV-2 PCR positive patients have a more pronounced biochemical prothrombotic phenotype and subsequently more frequent thrombotic complications. Elevated levels of established risk factors for thrombosis, in particular elevated levels of VWF and decreased levels of ADAMTS-13 which suggests endothelial activation, may be considered in the treatment of COVID-19.
